# Nutritional quality of feed resources used by smallholder dairy farmers in the Northern Province of Rwanda

**DOI:** 10.1007/s11250-025-04562-w

**Published:** 2025-07-18

**Authors:** Marie Anne Mukasafari, Mupenzi Mutimura, Ewa Wredle, Horacio Leandro Gonda

**Affiliations:** 1 Department of Animal Production, University of Rwanda, Box 57, Nyagatare, Rwanda; 2 Department of Applied Animal Science and Welfare, Swedish University of Agricultural Sciences, Box 7024, Uppsala, 750 07 Sweden; 3 Rwanda Agriculture and Animal Resources Development Board, Box 5016, Kigali, Rwanda

**Keywords:** East African countries, Forages, Crop residues, Fodder trees, Feed quality, Animal-source food

## Abstract

The study aimed to analyse both the type and quality of available feed resources used by smallholder dairy farmers in the Northern Province of Rwanda during the transition period from the short rainy season to the short dry season. A total of 218 feed samples were collected from 178 households. Twenty different feed types were identified and classified into: roadside vegetation (51%), cultivated grasses (45%), crop residues (23%), and cultivated trees (2%). Similar feeds from the same village and district were pooled, and the results from 175 feed samples were analysed for crude protein (CP), neutral detergent fibre (NDF), acid detergent fibre (ADF), and ash. Organic matter digestibility (OMD) and metabolisable energy (ME) content of the samples was calculated based on in vitro gas production at 24 h (GP_24_). Among all feed resources used, 42, 32, 12, and 9% of the households, respectively, minimally used feed such as Napier grass, assorted grasses, *Digitaria*, and maize stover. The CP content varied (*p* < 0.0001) between 4.4% in banana pseudo-stem and 34.8% in Elderberry tree. Only sweet potato vines, elderberry trees, and maize stover had significantly (*p* < 0.0001) higher OMD than the other feeds. The ME values ranged from 4.2 to 10.7 MJ/kg DM, with the lowest values reported for roadside vegetation (*Commelina* and gallant soldier), and the highest for Elderberry tree. Possible interventions such as training farmers in forage management and optimising the use of available feed resources, along with supplementing of higher-quality feed, can escalate productivity.

## Introduction

Livestock products serve several important purposes, including their contributions to food security, economic development, and cultural practices with high income from sales of animal products globally (Flax et al. 2023). Livestock contribute around 13% and 28% of global calories and protein, respectively, through direct consumption of animal-source foods (ASF) such as meat, eggs, or milk and provide essential nutrients for meeting humans’ nutritional requirements (McMahon 2016). Dairy products, for instance, are a valuable source of calcium, protein, and energy for young children, as well as pregnant or lactating women (McMahon 2016). Adequate intake of ASF, especially during pregnancy and the critical first 1000 days of life, has the greatest impact on children’s future growth and overall well-being (Martorell 2017). In most low-income countries such as Rwanda, the *per capita* consumption of ASF remains low (Balehegn et al. 2020). In Rwanda, even a low consumption of milk and milk products of 7.5 kg/capita/year, represents the main contributor to the food supply among other animal products, followed by meat and fisheries (Rwanda Food Balance Sheets 2017–2021) (NISR 2023). In smallholder farms, the amount of milk consumed at the household level is dependent on the amount produced at the household. In Rwanda the Self-Sufficient Ratio (SSR), the proportion of food consumed by a household that are produced at the same household, for animal products is 93.6% (Rwanda Food Balance Sheets 2017–2021). In relation to the low consumption of ASF, around 6.7% of children below the age of 5 experience chronic malnutrition with a 33.1% occurrence of stunting in Rwanda (National Institute of Statistics of Rwanda 2021).

It has been reported that milk yield at the majority of smallholder dairy farm is low, on average 3.2 L/cow/day (Kamanzi and Mapiye 2012; Klapwijk et al. 2014), limiting the availability of milk for human consumption, as well as the income of the household. Feed scarcity, both in terms of quantity and quality, is the main constraint that hinders livestock production (FAO 2018) as it limits the expression of the genetic potential of the livestock (Duguma 2020; Khan et al. 2009). In tropical countries, the availability of feed resources for livestock is highly dependent on climatic conditions including drought (Onono et al. 2013) and seasonality, particularly during the dry season (Kamanzi and Mapiye 2012; Bedada et al. 2021). Several studies have emphasised that factors such as forage types, phenological stage, and rainfall (Bezabih et al. 2014; Mutimura et al. 2017; Tefera et al. 2019), influence feed chemical composition, intake, digestibility, and therefore, milk production (Melaku et al. 2010; Kashongwe et al. 2017). During the dry season, feed resources are scarce and of low nutritional quality (Khan et al. 2009). In addition, animal breed can significantly affect milk yield, as Ankole crossbreds have the potential of producing more milk than pure Ankole (Manzi et al. 2020; Mukasafari et al. 2025).

A strategy to overcome feed shortage, and to subsequently improve animal productivity, is to cultivate improved forage varieties. However, the growing human population poses a significant challenge for poor smallholder farmers who face land shortage (Kamanzi and Mapiye 2012; Mutimura et al. 2019; Ndah et al. 2022). In addition, because many farmers are engaged in mixed crop-livestock production, a large portion of their land is usually allocated to crop production for human food, rather than for animal feed. Moreover, farmers have poor knowledge regarding pasture conservation and a lack of necessary facilities such as grass chopping and storage, as well as low accessibility to extension services (Kamanzi and Mapiye 2012; Ndah et al. 2022). Furthermore, limited land availability and restrictions on free grazing compel smallholder farmers to adopt zero grazing systems. While these systems allows for better control over feeding, require high input levels, particularly in terms of feeds which pose a significant challenge for resource-constrained households (Duguma 2022).

In East African countries, smallholder dairy farmers often rely solely on poor-quality natural forages and crop residues as basal diets for their dairy cattle. The limited availability of high-quality feed resources means that farmers are faced with the challenge of feeding their cattle, with whatever feed is accessible at minimal or no cost which typically results in very low productivity (Kamanzi and Mapiye 2012; Kashongwe et al. 2017). Majority of smallholder dairy farmers cannot supplement poor quality animal diets due to the shortage of quality feed and high cost of concentrates (Kashongwe et al. 2017). Efforts have been made to address feed scarcity and imbalanced diets to enhance animal performance (Khan et al. 2009) and to increase availability and consumption of ASF. To meet animal requirements, information regarding the nutritional value of the feed ingredients is needed (Khan et al. 2009). In Rwanda, information on the chemical composition of locally available feed resources for cattle is scarce. The current study aims to identify and evaluate the nutritional quality of locally available feed resources used by smallholder dairy farmers in northern Rwanda.

The present study is part of a bigger transdisciplinary project, which focuses on undernutrition in both children and their mothers. The project encompasses various fields of science including medicine and health sciences, agricultural and veterinary science, and geographical information systems by identifying a broader range of main risk factors contributing to stunting in children. Results of this study will enable opportunities for designing interventions and strategies aimed at improving feed management practices and, ultimately, enhancing the productivity and resilience of smallholder farmers in Rwanda, thereby addressing the issue of food insecurity.

## Materials and methods

### Study site

**Fig. 1:**
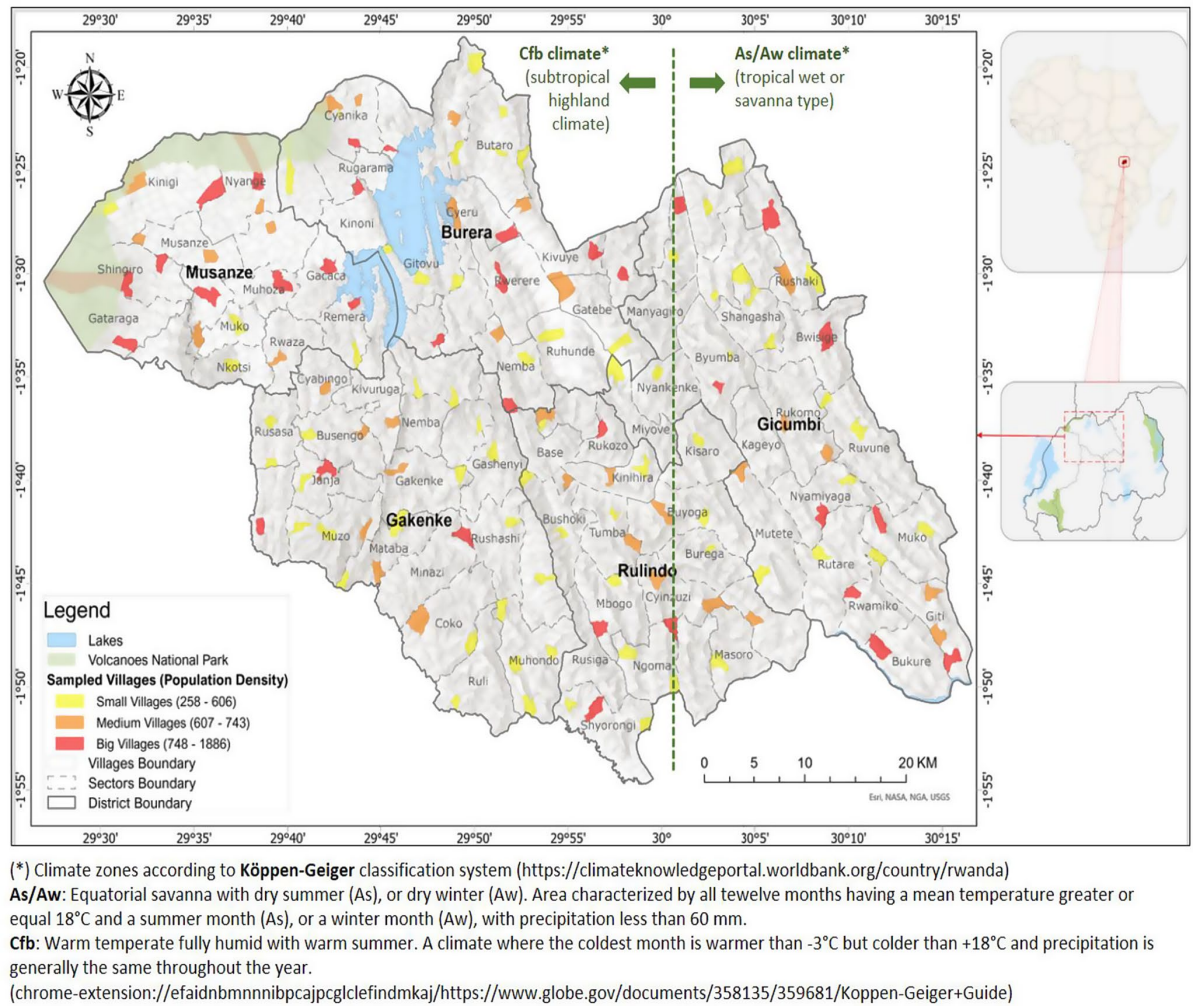
Map of the study sites, Northern Province, Rwanda (after edition by Kagoyire Clarisse)

This study was conducted at smallholder farms in the Northern Province of Rwanda, occasionally referred to as the Buberuka agro-ecological zone. The province comprises five districts, namely Burera, Gakenke, Gicumbi, Musanze, and Rulindo, with a total surface of 3,276 km^2^. According to the Köppen-Geiger climate classification, the district has areas being classified as a subtropical highland climate (Cfb), in Burera, Gakenke, Musanze, and Western part of Rulindo, while Gicumbi and Eastern part of Rulindo fall under the tropical wet or savannah climate (As/Aw) (Fig. 1). Rwanda generally experiences a bimodal rainfall pattern, resulting in four distinct seasons throughout the year, with the long rainy season from March to May, followed by a long dry season from June to mid-September. A shorter rainy season occurs from October to November, followed by a short dry season from December to February (https://www.expertafrica.com/rwanda/weather-and-climate). Northern Province has an estimated elevation of roughly 2,500 m above sea level, highest precipitation levels, cooler temperature averaging between 10 °C and 29 °C, and annual rainfall ranging from 1,000 to 1,400 mm. The native vegetation in this zone includes mountain climate zone, forests, natural grasslands, and wetlands, however, extensive agricultural activities have transformed much of the landscape. The study area is characterised by steep slopes and agriculture is the primary livelihood practice. To control soil erosion and improve crop yields, radical terracing is widely practiced within these highlands.

### Household selection and study design

The process of household selection involved multi-stage random sampling of villages and participants. In the first stage, 186 out of 2,743 villages were randomly selected using a geospatial grid system, representing 7% of all villages, followed by proportional sampling of households within each selected village (Utumatwishima et al. 2024). The number of households included in the study was estimated using the formula described by Kagoyire et al. (unpublished) after considering the proportion of stunting in the study area (41%; National Institute of Statistics of Rwanda 2021) (Eq. 1).

1 $$\:n=\frac{{Z}^{2}\:\text{x}\:p\:(1-p)}{{\epsilon\:}^{2}}\text{x}\:\text{D}\text{E}\text{F}\text{F}$$

Where: n is the sample size, Z is the z-score or the critical value associated with 95% confidence interval (1.96), *p* is the estimated proportion of stunting among children in the Northern Province (0.4), ε is the desired level of precision or margin of error (0.05), and DEFF is the design effect of 1.5 (to account for the increased variability that might be due to intra-cluster correlation).

The village leaders and Community Health Workers assisted in identifying households with mothers aged 18 and above, children between 1 months and 3 years of age, preferentially keeping a lactating cow. To guarantee the required sample size for the project on child undernutrition, if a household was unavailable on the day of data collection, a close neighbour was then selected provided that he/she had a similar living condition. A total of 630 households were initially interviewed; but 29 were excluded due to missing essential data. The final dataset included 601 households. Households were interviewed by using a comprehensive questionnaire, comprising a broader range of topics aimed to capture the various factors influencing child and maternal nutrition. The topics included were household characteristics; social support; reproductive history and antenatal care; child health; household dietary diversity; milk processing at the household; violence experience and controlling behaviour; mothers health; gender and decision-making in the household; and extensive agriculture and veterinary medicine. Data were collected only once at each household, from 29th November 2021 to 6th January 2022.

### Feed sample collection and preparation

Among all households, 178 (30%) had at least one lactating cow. From those households, a total of 218 feed samples, each weighing around 0.5 kg on fresh basis, were collected. Most households visited used just one type of feed rather than two types (Table 1). After sampling and during working days, feed samples were kept in labelled paper envelopes and stored in nearby district health centres. During storage before drying, samples were allowed to wilt at room temperature avoiding any deterioration due to moist content. By the end of the week, samples were moved to an air-drying room at the University of Rwanda, Busogo Campus to be subsequently transferred to the Rwanda Agriculture and Animal Resources Development Board (RAB) laboratory at Rubona Station for chemical analysis. Feed samples were oven-dried at 65 °C until reaching a constant weight using electronic weighing scale (Adam Equipment.com; Model PW254: UK). Samples of the same feeds and from the same village were combined into one sample. After combining, a total of 175 out of 218 collected feed samples were ground to pass through a 1 mm sieve and kept in airtight containers until analysis (Table 1).

**Table 1:** Number of samples collected from the households with lactating cattle at different districts in the Northern Province of Rwanda

District	Household visited (*n* = 178)	Feed samples collected (*n* = 218)	Ratio of feeds used per household	Feed samples after combining (*n* = 175)
Burera	32	40	1.25	34
Gakenke	18	27	1.50	18
Gicumbi	54	60	1.11	44
Musanze	40	52	1.30	42
Rulindo	34	39	1.15	37

### Chemical analysis

All 175 combined samples were analysed for crude protein (CP), neutral detergent fibre (NDF), acid detergent fibre (ADF), and ash. Determinations of CP and ash were carried out according to AOAC (2023), and NDF and ADF fractions according to Goering and Van Soest (1970).

### In vitro gas production

Prior to the in vitro gas production, feed samples were further grouped. Samples of the same feed resources from different districts were combined based on their NDF levels. This categorisation resulted in 43 out of 175 feed samples being selected for in vitro gas production.

Aliquots of 0.2 g of each sample were weighed into 100-mL airtight glass syringes in triplicate (Samco, Eterna-Matic, Italy). Within 2 h before each incubation, rumen fluid (RF) was collected from slaughtered cattle at Matyazo slaughterhouse in Huye district, Rwanda. Within 15 min after the animal was slaughtered, samples of RF were squeezed, transferred to a pre-warmed thermos, immediately sealed, and delivered to the laboratory. Upon arrival, RF was pooled and filtered using two layers of cheesecloth, then poured into pre-warmed buffer solutions prepared according to Osuji et al. (1993) in a ratio of 1:2 (v/v) with continuous CO_2_ flushing. Using a veterinary drenching cannon (HSW, ROUX-REVOLVER, Germany), approximately 30mL of anaerobic buffered RF was pipetted and thoroughly poured into the glass syringes for incubation in an oven at 39 ± 1 ^0^C for 72 h (Mammart, D_0606´: Model600, Germany). The gas production (GP) was visually recorded by reading the displacement of the plungers at different times of 0, 2, 6, 8, 12, 18, 24, 36, 48, 60, and 72 h. At each reading, syringes were gently shaken. Two to three blank syringes, containing only buffered rumen fluid were also incubated at each of the 4 runs.

### Data handling

The collective gas produced was calculated for each glass syringe by computing the difference of gas recorded at a certain time (t_i_) and an average of blank syringe (t_0_). Cumulative gas production (GP) data were adjusted at the model proposed by Ørskov & Mcdonald. (1979) (Eq. 2):

2 $$\:GP\left(t\right)=a+b(1-{e}^{-ct})$$

Where: GP _(*t*)_ is the cumulative gas produced at time *t*, ‘*a*’ is gas produced by the soluble fraction (mL), ‘*b*’ is gas produced by insoluble but potentially degradable feed, ‘*c*’ is constant rate of GP (mL/h), and ‘*t*’ is half-time of gas production (h). Organic matter digestibility (OMD) and metabolisable energy (ME) were estimated on the basis of the mean of gas volume produced at 24 h (G_24_) according to Menke et al. (1979), as follows, Eqs. 3 and 4:

3 $$\:\text{O}\text{M}\text{D}\left(\text{\%}\right)=148.8+{8.89\:\text{G}\text{P}}_{24}+4.5\text{C}\text{P}+0.651\text{A}$$

Where: G_24_ is the cumulative gas volume at 24 h after inoculation, CP is the crude protein (%), and A is the ash content (%).

4 $$\:\text{M}\text{E}\left(\frac{\text{M}\text{J}}{\text{k}\text{g}}\text{D}\text{M}\right)=2.2+0.136{\:\text{G}\text{P}}_{24}+0.057\text{C}\text{P}+{0.0029\text{C}\text{P}}^{2}$$

Where: G_24_ is the cumulative gas volume at 24 h after inoculation, and CP is the crude protein (g/kg DM).

### Data analysis

Data on chemical composition and in vitro cumulative gas production were subjected to analysis of variance (ANOVA) using the Statistical Analysis System version 9.4 (SAS Institute Inc., 2016). Significant differences in feed composition parameters among continuous variables were addressed by using GLM procedures of SAS and differences among means were determined by the Student-Newman-Keuls (SNK) test at 5% significance level. The model used for the analyses of nutritional quality was: $\:Yij=\:\mu\:+bi+eij$

where Yij = mean for response variable; µ = overall mean; bi = the effect of the *i*^*th*^ feed samples and eij = effect of the random error of the *i*^*th*^ feed samples.

## Results

### Household characteristics

In the study area, 70% of the surveyed households were landless, with the highest number being in the Rulindo District (Table 2). A smaller number of households owned between 1 and 5 ha of land, particularly in the Musanze and Gakenke districts, while very few households owned more than 5 ha. Regarding livestock ownership, on average 64% of households owned cattle. The Gicumbi district had the highest number of households with cattle (76%). In addition to cattle, farmers also owned goats, poultry, pigs, and rabbits (Table 2). Households in the Gicumbi district commonly keep both goats and pigs, while in the Gakenke district, pigs were more common than in other districts. Most households across all five districts fall into the lowest income category 1(< 36,000 Frw), with Gicumbi having the greatest percentage compared to Burera districts. A much smaller number of households fall into income category 2 and 3, while very few households reported being unsure of their income, with only 2% or less in most districts.

**Table 2:** Household social-economic characteristics in the Northern Province of Rwanda

Variable	District				
	Burera (*n* = 129)	Gakenke (*n* = 130)	Gicumbi (*n* = 144)	Musanze (*n* = 96)	Rulindo (*n* = 102)
**Farm size (%)**					
Unclassified	4	3	0	3	8
Landless	68	67	69	72	74
< 1 ha	6	5	10	6	7
1–5 ha	20	21	19	19	9
> 5 ha	2	4	2	0	2
**Household with cow (%)**	52	64	75	53	75
**Household with other livestock (%)**					
Goat	18	25	30	7	16
Poultry	18	22	28	16	22
Pig	16	36	12	13	21
Rabbit	3	6	6	2	16
Sheep	22	15	9	21	9
**Feeding system for cattle (%)** ^*^					
Zero grazing	93	100	98	47	84
Semi-zero grazing^#^	7	0	2	50	16
Grazing	0	0	0	3	0
**Major source of forage (%)** ^*^^					
Collected (*n* = 144)	100	94	92	83	94
Planted (*n* = 132)	79	100	94	57	94
Both practices (*n* = 86)	31	94	50	63	56
**Household income category (%)** ^**##**^					
1	77	85	88	79	86
2	19	8	5	18	11
3	4	5	6	3	1
Dont know	0	2	1	0	2

^*^Frequency for those owning lactating cow; ^#^Includes tethering; ^^^each variable was analysed individually; ^##^household income category: 1 = less than 36,000 Frw (Rwandan Francs); 2 = income between 36,000 to 99,000 Frw; 3 = above 100,000 Frw [1 USD = 1, 325 Frw] (September 2024)

### Feed types

In this study, a total of 20 different feeds were identified. However, only a few were used by a significant number of households. Thus, while Napier grass, assorted grass, *Digitaria*, and maize stover were used by 42, 32, 12, and 9% of households, respectively, all other feeds were used by just less than 5% of households (Table 3). Feed types were classified into four distinct categories, namely roadside vegetation, cultivated grasses, crop residues, and fodder trees. Among the districts, certain differences in the use of the available feed resources were observed. Across all districts, 51% of the farmers used roadside vegetation, followed by cultivated grasses (45%), and crop residues (23%) as their primary feed resources. The use of fodder trees was very low (2.3%), and exclusive for households in Musanze district. Farmers in Burera district used less roadside vegetation and more cultivated grasses than farmers in the other districts. The use of cultivated grasses was great in Burera and Gakenke districts, where nearly 75% of the households used Napier grass. Musanze was the district where the use of cultivated grasses was the lowest, and crop residues used was the greatest. Musanze, Burera, and Gicumbi exhibited a greater diversity of feed resources than Rulindo and Gakenke.

**Table 3:** Locally available feed resources used by farmers during the period of short wet season in the Northern Province of Rwanda

Feed resources		Districts					% of HH	Fed as (% of HH)	
Common name	Scientific name	Burera 32 HH	Gakenke 18 HH	Gicumbi 54 HH	Musanze 40 HH	Rulindo 34 HH		Alone	Mixed
**Roadside vegetation**									
Assorted grass	NA	5	1	23	16	12	*32*	*37*	*63*
*Commelina*	*Commelina communis*	1	-	1	-	-	*1*	*50*	*50*
*Digitaria*	*Digitaria sp*	3	7	3	3	6	*13*	*36*	*64*
*Eragrostis*	*Eragrostis sp*	-	2	1	-	2	*3*	*60*	*40*
Gallant soldier	*Galinsoga parviflora*	1	-	-	4	-	*3*	*60*	*40*
*% of households*		*31*	*56*	*52*	*58*	*59*	*-*	*-*	*-*
**Cultivated grasses**									
*Brachiaria*	*Urochloa sp.*	1	-	-	1	-	*1*	*0*	*100*
Kikuyu grass	*Cenchrus clandestinus*	2	-	1	-	-	*2*	*33*	*67*
Napier grass	*Cenchrus purpureus* ^*#*^	19	14	24	6	11	*42*	*38*	*62*
Setaria grass	*Setaria sp.*	2	-	-	1	-	*2*	*100*	*0*
*% of households*		*75*	*78*	*46*	*20*	*32*	*-*	*-*	*-*
**Crop residues**									
Banana leaves	*Musa sp.*	-	-	1	-	-	*1*	*0*	*100*
Banana pseudostem	*Musa sp.*	-	2	2	2	2	*5*	*25*	*75*
Bean haulms	*Phaseolus vulgaris*	-	-	2	1	2	*3*	*20*	*80*
Irish potato haulms	*Solanum tuberosum*	-	-	-	3	-	*2*	*100*	*0*
Maize stover	*Zea mays*	3	1	1	10	1	*9*	*56*	*44*
Peas haulms	*Lathyrus oleraceus*	1	-	-	-	-	*1*	*100*	*0*
Soybean haulms	*Glycine max*	1	-	-	-	-	*1*	*0*	*100*
Sugarcane leaves	*Saccharum officinarum*	-	-	-	-	1	*1*	*0*	*100*
Sweat potato vines	*Ipomoea batatas*	1	-	1	1	2	*3*	*60*	*40*
*% of households*		*19*	*17*	*13*	*45*	*24*	*-*	*-*	*-*
**Fodder trees**									
*Calliandra*	*Calliandra calothyrsus*	-	-	-	3	-	*2*	*0*	*100*
Elderberry	*Sambucus nigra*	-	-	-	1	-	*1*	*0*	*100*
*% of households*		*0*	*0*	*0*	*10*	*0*	*-*	*-*	*-*
**Total feed types per district**		12	6	11	13	9		**-**	**-**

^HH^ households, ^NA^ not available, ^−^Feed resources not used in the district, ^#^Also known as *Pennisetum purpureum*

Within roadside vegetation, assorted grass was more frequently used in all districts except in Gakenke, at which *Digitaria* was most used. Napier grass was the most used of all cultivated grasses in all districts, and the only one used in Rulindo and Gakenke. Farmers in Gakenke use exclusively Napier grass, whereas those in Burera used Kikuyu grass, Napier grass, *Brachiaria*, and *Setaria*. Across all districts, common crop residues used were maize stover, sweet potatoes vines, banana pseudo-stem, and bean haulms. Musanze was the exception, where maize stover was used by almost 60% of the households that used crop residues, whilst in the other four districts maize stover was used by only a few households. Fodder trees were exclusively used in Musanze district but only by 10% of the households. Both species, *Calliandra* and Elderberry trees, were fed mixed with other feeds. Most of the different feeds were fed either alone or mixed with others in a similar proportion. Some exceptions were assorted grasses, Napier grass, and banana pseudo-stem, which were more commonly fed in combination with other feed.

### Chemical composition of available feed resources

There was a noticeable variation in the chemical composition of the feed resources utilised by smallholder farmers (Table 4). The CP content ranged from 4.4% in banana pseudo-stem to 34.8% in Elderberry trees. Fodder trees consistently exhibited significantly (*p* < 0.0001) greater levels of CP content compared to other feed resources. There were no significant differences observed in CP content among feeds categorised in roadside vegetation and cultivated grasses. The CP content in crop residues was more variable, where Irish potato haulms had the greatest CP content (*p* < 0.0001). Concerning the average structural constituents, NDF was slightly greater in roadside vegetation especially in *Eragrostis* compared to gallant soldier. Despite numerical differences, there were no significant differences in the content of NDF among all feed resources except between *Eragrostis*, Irish potato haulms, and Elderberry trees. This was also observed for ADF, with the exception of Kikuyu grass which had a lower ADF content (*p* < 0.001) than *Eragrostis* and sugarcane leaves. The average ash content ranged from 6.3 to 23.1%, with gallant soldier having the greatest ash content among the feeds, which significantly surpassed that of *Eragrostis*. Among the 20 feed resources, only sweet potato vines, Elderberry trees, and maize stover had significantly (*p* < 0.0001) greater OMD compared to other feed resources (Table 5). Contrastingly, the lowest OMD value was observed in *Commelina*. The ME values ranged from 4.2 to 10.7 MJ/kg DM, with the lowest values reported in roadside vegetation, particularly in *Commelina* and gallant soldier. The ME values of Elderberry trees was significantly greater (*p* < 0.0001) than all other feed resources.

**Table 4:** Chemical composition of locally available feed resources used in smallholder dairy farms in the Northern Province of Rwanda

Feed resources (# of samples)	Feed composition parameters (%)			
	CP	NDF	ADF	Ash
**Roadside vegetation**				
Assorted grass (*n* = 45)	12.2^de^	52.9^ab^	35.9^abc^	16.3^ab^
*Commelina* (*n* = 2)	9.2^de^	52.5^ab^	30.2^abc^	13.1^ab^
*Digitaria* (*n* = 20)	9.6^de^	60.4^ab^	38.6^abc^	12.7^ab^
*Eragrostis* (*n* = 4)	7.4^de^	70.4^a^	45.5^ab^	6.3^b^
Gallant soldier (*n* = 2)	8.8^de^	48.7^ab^	35.3^abc^	23.1^a^
**Cultivated grasses**				
*Brachiaria* (*n* = 2)	10.3^de^	56.1^ab^	40.8^abc^	17.4^ab^
Kikuyu grass (*n* = 3)	12.1^de^	35.4^ab^	21.5^c^	16.2^ab^
Napier grass (*n* = 53)	10.2^de^	60^ab^	37.0^abc^	16.4^ab^
*Setaria* (*n* = 3)	9.3^de^	63^ab^	37.9^abc^	17.2^ab^
**Crop residues**				
Banana leaves (*n* = 1)	13.0^de^	41.2^ab^	34.1^abc^	12.4^ab^
Banana pseudo-stem (*n* = 7)	4.4^e^	59.9^ab^	41.6^abc^	14.2^ab^
Bean haulms (*n* = 5)	8.6^de^	60^ab^	40.6^abc^	8.7^ab^
Irish potato haulms (*n* = 3)	19.2^c^	30.3^b^	25.9^bc^	21.0^ab^
Maize stover (*n* = 14)	11.0^de^	54.8^ab^	34.5^abc^	12.7^ab^
Peas haulms (*n* = 1)	13.8^d^	65.6^ab^	46.6^ab^	7.9^ab^
Soybean haulms (*n* = 1)	7.3^de^	57.4^ab^	28.4^abc^	8.2^ab^
Sugarcane leaves (*n* = 1)	10.9^de^	69.3^ab^	50.2^a^	10.1^ab^
Sweet potato vines (*n* = 5)	10.1^de^	45.1^ab^	33.1^abc^	12.7^ab^
**Cultivated trees**				
*Calliandra* (*n* = 2)	24.6^b^	49.8^ab^	35.5^abc^	6.7^b^
Elderberry tree (*n* = 1)	34.8^a^	30.4^b^	25.2^bc^	10.1^ab^
RMSE	2.97	12.14	7.21	4.71
*P-value*	*0.0001*	*0.002*	*0.001*	*0.0001*

^a−e^ Means with different superscripts in the column are significantly different at least at *p* < 0.05. DM = Dry matter, CP = Crude protein, NDF = Neutral detergent fibre, ADF = Acid detergent fibre, n = number of feeds samples

**Table 5:** In vitro gas production parameters, organic matter digestibility (OMD) and metabolisable energy (ME) of the feed resources used by smallholder farmers in the Northern Province of Rwanda

Feed resources	In vitro gas production parameters					
	GP_72_	GP_24_	C	T_1/2_	OMD	ME
	mL/0.2 g DM	mL/0.2 g DM	mL/hr	hr	(%)	MJ/kg DM
**Roadside vegetation**						
Assorted grass	37.5^abc^	20.5^a^	0.02^b^	27.9^abcde^	37.6^cde^	5.8^cde^
*Commelina*	28.4^abcdef^	8.3^b^	0.02^b^	36.8^a^	27.8^f^	4.2^f^
*Digitaria*	32.5^abcdef^	14.3^ab^	0.02^b^	31.0^abcd^	33.9^cdef^	5.1^def^
*Eragrostis*	34.8^abcd^	15.0^ab^	0.02^b^	33.6^abc^	31.8^def^	4.8^ef^
Gallant soldier	20.0^def^	14.5^ab^	0.04^ab^	22.1^bcde^	29.1^ef^	4.3^f^
**Cultivated grasses**						
*Brachiaria*	35.0^abcd^	14.5^ab^	0.02^b^	35.1^ab^	34.0^cdef^	5.1^def^
Kikuyu grass	28.8^abcdef^	14.7^ab^	0.04^ab^	28.6^abcde^	34.4^cdef^	5.3^cdef^
Napier grass	39^ab^	19.3^ab^	0.02^b^	26.2^abcde^	37.3^cde^	5.7^cde^
Setaria grass	33.2^abcde^	17.7^ab^	0.03^b^	27.6^abcde^	35.8^cdef^	5.4^cdef^
**Crop residues**						
Banana leaves	18.7^ef^	9.7^b^	0.02^b^	33.6^abc^	31.3^def^	4.9^ef^
Banana pseudo-stem	37.4^abc^	16.9^ab^	0.03^b^	21.2^bcde^	34^cdef^	5.0^ef^
Bean haulms	39.6^ab^	21.2^ab^	0.02^b^	26.1^abcde^	36.8^cde^	5.6^cde^
Irish potato haulms	18.8^ef^	14.3^ab^	0.06^a^	15.9^e^	37.9^cde^	6.3^bcd^
Maize stover	43.6^a^	28.9^a^	0.04^ab^	19.3^cde^	46.1^ab^	7.1^b^
Peas haulms	31.5^abcdef^	22.0^ab^	0.05^ab^	17.4^de^	41.2^bc^	6.5^bc^
Soybean haulms	22.7^cdef^	15^ab^	0.05^ab^	19.7^cde^	32.1^def^	4.8^ef^
Sugarcane leaves	33.5^abcde^	21.7^ab^	0.05^ab^	21.3^bcde^	39.7^cd^	6.1^bcde^
Sweet potato vines	39.7^ab^	29.2^a^	0.05^ab^	15.4^e^	47.4^ab^	7.2^b^
**Cultivated trees**						
*Calliandra*	17.4^f^	11.5^ab^	0.02^b^	17.9^de^	37.5^cde^	7.1^b^
Elderberry tree	25.3^bcdef^	21.7^ab^	0.06^a^	13.9^e^	50.5^a^	10.7^a^
RMSE	4.99	5.9	0.01	4.91	5.14	0.64
P-value	< 0.0001	0.008	< 0.0001	0.0001	< 0.0001	< 0.0001

^a−f^ Means with different superscripts in the column are significantly different at least at *p* < 0.05; GP_72_ = total gas production at time 72 h; GP_24_ = gas production at 24 h; C = constant rate of gas production; T_½_= time required to generate 50% of total GP

### In vitro gas production

Total GP of the feed resources was significantly different (*p* < 0.0001) and ranged between 16.2 and 40.8 mL/0.2 g DM (Table 5). Gallant soldier, banana leaves, Irish potato haulms, soybean haulms, *Calliandra*, and Elderberry trees had lower GP (< 25 mL/0.2 g DM; *p* < 0.001) than all other feeds. Numerically, the greatest GP values (> 35 mL/0.2 g DM) corresponded to assorted grass, Napier grass, banana pseudo-stem, maize stover, peas haulms, and sweet potato vines. Values of the constant rate of GP (C) ranged between 0.018 and 0.058 h, with the lowest value corresponding to banana leaves and the greatest to Elderberry tree. The time taken to achieve 50% of total GP (T_½_) ranged between 13.9 and 36.8 h. Feeds that showed the shortest times and therefore, faster fermentation, were banana leaves, maize stover, and Elderberry tree, and the longer times corresponded to *Commelina* and *Brachiaria* (Table 5).

**Fig. 2:**
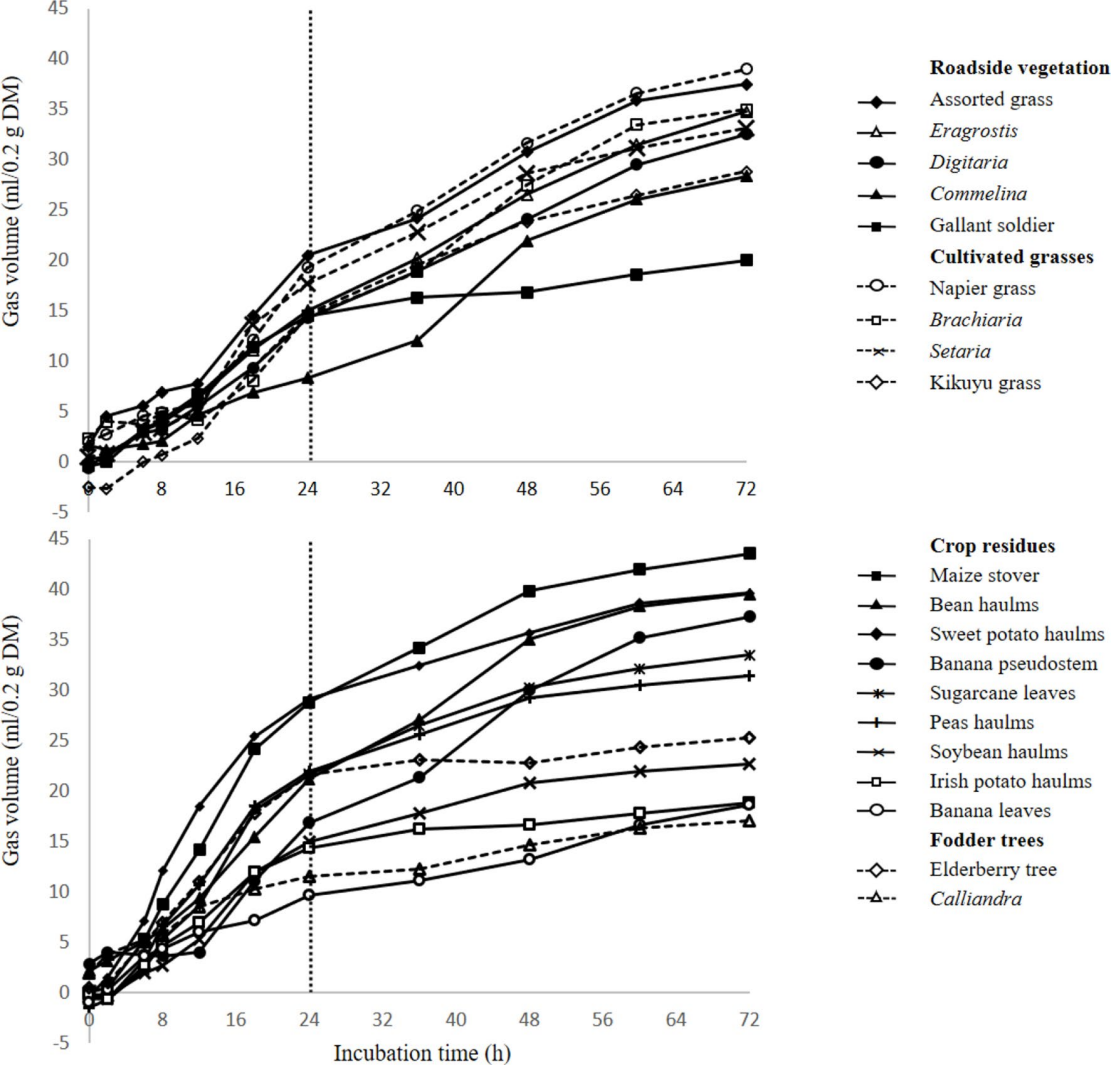
In vitro gas production of feed resources used by smallholder farmers in the Northern Province of Rwanda incubated within 72 h

The cumulative gas production profile at different incubation time is displayed in Fig. 2. Across all feed categories, gas production started after initial incubation, and showed an increase between 24 h and 72 h.

## Discussion

Rwanda is a landlocked country, located south of the Equator in East-Central Africa between latitudes 1°04’ and 2°51’ south and longitudes 28°45’ and 31°15’ east. The land has a total area of 26,340 km², with a population of approximately 14.5 million people, making it the most densely populated country in the world. A considerable share of the inhabitants (82%) belongs to the rural population. Mixed crop-livestock production is the predominant farming system in Rwanda wherein most of the crop residues used were directly obtained from farming activities or neighbours (Klapwijk et al. 2014). Due to land scarcity, free grazing is prohibited in the country. Instead, farmers across all districts practice zero grazing, especially in Gakenke, Gicumbi, and Burera. However, households in Musanze are the exception, where free grazing is still practiced. Farmers in the study area do not conserve feeds as hay or silage, rather, they rely on a cut-and-carry system to collect forages from their farms or other locations. To cope with the feed shortage, some farmers grow forages on terraces alongside their crops, though not all have adopted this practice (Kamanzi and Mapiye 2012).

This study aimed to generate information on the distribution and nutritional quality of locally available feed resources used by smallholder dairy farmers in the Northern Province of Rwanda. The available feeds utilised by smallholder farmers were grouped into four categories, namely: roadside vegetation, cultivated grass, crop residue, and fodder trees. Feeds reported in this study are similar to those found in Southwestern Rwanda (Klapwijk et al. 2014) and other East African countries (Kiggundu et al. 2014; Mutimura et al. 2015; Maleko et al. 2018; Mamo et al. 2023). Variation of available feed resources, both in quantity and quality, is dependent on various factors such as land owned to grow forages, seasonality, accessibility (Mutimura et al. 2019; Tefera et al. 2019; Fentahun et al. 2020), and forage phenological stage (Mutimura et al. 2015), among others. In East African countries, including Rwanda with its high population density, farmers face land shortage, which resultantly inhibits their ability to grow forages for their livestock. In our study, most farmers were landless, with some owning 1 to 5 ha of land. The lack of enough land causes most farmers in Rwanda, similar to many other developing countries, to prioritise growing food crops over forages (Gebremariam and Belay 2016), especially when food security is a major concern. If the opportunity of cultivating forages is there, it would occur on terraces alongside the crop-growing areas (Kamanzi and Mapiye 2012; Crovetto et al. 2022). It was found that Napier grass was the most commonly cultivated grass in Gicumbi compared to the other districts. Farmers generally prefer Napier grass due to its year-round yield, its ability to tolerate tropical conditions, reduce soil erosion, and its resilience (Khan et al. 2011). With regard to fodder trees, the least used feed resource as seen by Klapwijk et al. (2014), it can be said that they appear as a good protein rich supplementary feed.

Roadside vegetation and crop residues were the alternative feed resources collected in a cut-and-carry system from either their farms, communal lands, or roadsides. Among roadside vegetation, assorted grass and *Digitaria* were abundantly used as a feed across all districts. In the current study, data collection took place during the transition from the short rainy season to the short dry season (November to January), a period characterised by limited rainfall. Various researchers have reported that during the dry season, farmers predominantly rely on crop residues to feed their livestock (Kamanzi and Mapiye 2012; Fentahun et al. 2020; Mamo et al. 2023). This practice is common in many rural and peri-urban areas in East African countries, where farmers rely on both their own agricultural by-products and a collaborative community. In our study, maize stover was the most used crop residue by farmers in Musanze, as reported for Southern Rwanda (Kamanzi and Mapiye 2012). However, Kiggundu et al. (2014) and Klapwijk et al. (2014) reported that maize stover was the least used feed by farmers in their studies. This is likely due to the fact that, almost all of the maize stover was left in the fields after the harvest of the cob for mulching or used as firewood (Kiggundu et al. 2014). Similar to our findings, Klapwijk et al. (2014) highlighted that banana pseudo-stem was more frequently used than banana leaves. Further, in Uganda banana plantains are a staple food and their by-products, such as pseudo-stems and peels, are more commonly used than leaves (Lumu et al. 2013).

Unsurprisingly, the chemical composition of the different feed resources varied considerably. In general, values of CP and fibre in the current study were similar to those provided by Feedipedia (https://feedipedia.org), and other reports (Klapwijk et al. 2014; Mutimura et al. 2015; Crovetto et al. 2022). The CP content of roadside vegetation and cultivated grasses was similar among the different species of forages, and above the minimum CP content capable of reducing rumen microbial activity (Van Soest 1994). The CP content of the most commonly used feed resources, Napier grass and assorted grass, falls within the range reported by Nyaata et al. (2000) in Kenya, although they were slightly greater than those reported by Mutimura et al. (2015). The CP content in banana pseudo-stem samples was the lowest of all feed resources, but greater than the value reported by Klapwijk et al. (2014) and similar to values found by Mutimura et al. (2015). In contrast to banana pseudo-stem, Irish potato haulms, with a CP content close to 20%, appeared to be a potential protein supplement in combination with other energy rich feeds. The same can be said for Elderberry and *Calliandra* trees which had a CP > 20%. The greater CP contents in Elderberry tree, although lower than the one observed in the present study, were reported by Młynarczyk et al. (2018) and Castillo et al. (2019). Different CP content in samples of Elderberry tree can be explained by the fact that CP content differs among the different parts of the plant i.e., leaves, flowers, or fruits (Młynarczyk et al. 2018). Mutimura et al. (2015) and Klapwijk et al. (2014) also found a greater CP content in samples of *Calliandra* tree.

Assorted grass and Napier grass were, by far, the most commonly used feed resources within the categories of roadside and cultivated grasses, respectively. Assorted grasses were shown to have a numerically lower mean NDF content than Napier grass, as 67% of the samples collected had NDF content < 60%, whilst 64% of the samples of Napier grass had an NDF content > 57% (data not presented). This suggests that there is room for improving the nutritional quality of the cultivated Napier grass by cutting it at earlier stage of growth (Mutimura et al. 2017). The nutritional quality of forages decreases as they progress from the vegetative to the mature stage, with young forages being more digestible and greater in protein. As forages mature, fibre content increases, reducing DM intake, digestibility, and overall nutritive value for livestock (Mamo et al. 2023). In general terms, according to Kitaba and Tamir (2007), feed resources with an fibre content less than 45% can be classified as greater-quality, those with a content between 45% and 65% can be classified as medium-quality, and feeds with a fibre content > 65% can be considered to be of poor quality.

Mutimura et al. (2015), following the same protocol as in the current study but using a rumen fistulised cow as a donor of rumen fluid, reported a greater ME value, on average 14% greater, than those reported in this study for samples of *Commelina*, Napier grass, *Setaria*, banana leaves and pseudo stem, Irish potato haulms, sweet potato vines, and maize stover. Various factors such as the conditions on which rumen fluid was collected, and/or variation in the phenological stage of the feeds, could explain the observed differences (Sallam et al. 2007). Nonetheless, OMD and ME content were relatively similar among roadside vegetation and cultivated grasses. Among crop residues, maize stover and sweet potato vines had the greatest OMD and ME values. Across all feed resources, ME values of banana leaves, *Calliandra*, and *Commelina* were lower compared to the report in Sub Saharan Africa database (https://feedsdatabase.ilri.org/). The lowest ME values reported in these feeds may be explained by the method used for OMD determination. In the present study, OMD was based in gas production, and the presence of condensed tannins and other secondary metabolites in banana leaves and legumes can interfere with rumen microbial activity (Oliveira et al. 2014), reducing the amount of gas produced and, therefore, the calculated OMD. Similarly, phenolic compounds and tannins presents in Elderberry tree (Młynarczyk et al. 2018) may explain its low gas production. However, due to its high CP content, Elderberry tree has the highest OMD and ME values.

Even when the ME content of most of the feed resources used by smallholder dairy farmers is low, it could be argued that it is enough to sustain a slightly greater milk yield than the average of 3.5–5 L/cow/d (Kamanzi and Mapiye 2012; Mwendia et al. 2018; Mukasafari et al. 2025). It is possible that, not only could the quality of the feed resources be a limiting factor, but also the amount of feed that it is being offered to the cows is insufficient. Unfortunately, to our knowledge, there is no information within the literature about the amount of feed and the feeding practices, as frequency of feeding, usually used by the smallholder farmers, nor about the water supply in quantity and quality. Smallholder dairy farmers in East African countries face a major challenge of seasonal water shortages during the dry season, which restricts water availability for livestock and negatively affects the milk production of improved cattle breeds with greater water needs (Bosire et al. 2019).

## Conclusions

In conclusion, this study presented valuable insights into both the availability and quality of local feed resources used by smallholder farmers in Northern Rwanda. Feeds distribution among smallholder dairy farms varies. Most farmers rely on roadside vegetation and cultivated grass fed either alone or in combination. All feed resources, except banana pseudo-stem, provide an amount of CP that does not limit microbial activity. However, ME content may be a limiting factor. Harvesting cultivated forages at early stage of growth would allow them to increase their ME and CP contents. Elderberry tree, *Calliandra*, and Irish potato haulms have the potential to be used as protein-rich supplements to improve the nutritional quality of low-quality basal diets, such as crop residues or roadside vegetation. To address the challenges linked to feed quantity and quality in smallholder farms, possible interventions such as training farmers in forage management and optimising the use of available feed resources, along with supplementing of greater-quality feed, can accelerate productivity. These efforts hold significant potential to enhance human well-being in the long-term.

## Data Availability

Original research data of this manuscript is available from the corresponding author on reasonable request.
